# Transcriptome sequencing supports a conservation of macrophage polarization in fish

**DOI:** 10.1038/s41598-020-70248-y

**Published:** 2020-08-10

**Authors:** Annelieke S. Wentzel, Jules Petit, Wouter G. van Veen, Inge Rosenbek Fink, Marleen H. Scheer, M. Carla Piazzon, Maria Forlenza, Herman P. Spaink, Geert F. Wiegertjes

**Affiliations:** 1 Cell Biology and Immunology Group, Aquaculture and Fisheries Group, Wageningen University and Research, De Elst 1, 6708 WD Wageningen, The Netherlands; 2 Aquaculture and Fisheries Group, Wageningen University and Research, De Elst 1, 6708 WD Wageningen, The Netherlands; 3 Experimental Zoology Group, Wageningen University and Research, De Elst 1, 6708 WD Wageningen, The Netherlands; 4 Fish Pathology Group, Institute of Aquaculture Torre de La Sal (IATS-CSIC), 12595 Ribera de Cabanes, Castellón Spain; 5 Institute of Biology, Leiden University, Einsteinweg 55, 2332 CC Leiden, The Netherlands

**Keywords:** Monocytes and macrophages, Immunogenetics, Evolutionary biology, Gene expression profiling, RNA sequencing, Innate immunity

## Abstract

Mammalian macrophages can adopt polarization states that, depending on the exact stimuli present in their extracellular environment, can lead to very different functions. Although these different polarization states have been shown primarily for macrophages of humans and mice, it is likely that polarized macrophages with corresponding phenotypes exist across mammals. Evidence of functional conservation in macrophages from teleost fish suggests that the same, or at least comparable polarization states should also be present in teleosts. However, corresponding transcriptional profiles of marker genes have not been reported thus far. In this study we confirm that macrophages from common carp can polarize into M1- and M2 phenotypes with conserved functions and corresponding transcriptional profiles compared to mammalian macrophages. Carp M1 macrophages show increased production of nitric oxide and a transcriptional profile with increased pro-inflammatory cytokines and mediators, including *il6, il12* and *saa*. Carp M2 macrophages show increased arginase activity and a transcriptional profile with increased anti-inflammatory mediators, including *cyr61*, *timp2b* and *tgm2b*. Our RNA sequencing approach allowed us to list, in an unbiased manner, markers discriminating between M1 and M2 macrophages of teleost fish. We discuss the importance of our findings for the evaluation of immunostimulants for aquaculture and for the identification of gene targets to generate transgenic zebrafish for detailed studies on M1 and M2 macrophages. Above all, we discuss the striking degree of evolutionary conservation of macrophage polarization in a lower vertebrate.

## Introduction

Depending on stimuli present in their extracellular environment, mammalian macrophages can adopt polarization states that can exert very different, sometimes opposite, functions. These opposite functional differences were initially referred to as the M1/M2 paradigm^1^, in which M1 macrophages exert pro-inflammatory activities driven by Th1 cytokines as opposed to M2 macrophages that would be driven by Th2 cytokines and be involved in anti-inflammatory responses. This paradigm is primarily based on arginine metabolism, as inflammatory M1 macrophages metabolize arginine to produce anti-microbial nitric oxide (NO) while anti-inflammatory M2 macrophages utilize the same arginine to produce proline and polyamines required for cell proliferation and tissue generation. In more recent studies, the M1/M2 paradigm has been refined to include at least nine distinct macrophage activation states^2^ or define M1 and M2 macrophages at the opposite ends of an entire spectrum of activation states^2-5^. Different macrophage polarization states have been studied in detail in mice and men, however it remains unclear to what extend these polarized phenotypes are conserved in non-mammalian species. Although considerable differences exist between polarized macrophages of mammals including mice and men^6-8^, their M1 and M2 macrophages display comparable core phenotypes and it is likely that polarized macrophages with corresponding core phenotypes exist throughout mammals. Based on our previous work^9,10^ we hypothesize that these comparable basic phenotypes would also be displayed by macrophages of common carp (*Cyprinus carpio*), a teleost species that shared the last tetrapod’s common ancestor more than 350 million years ago and is an important species for aquaculture^11^.

In fish, the ability of macrophages to polarize towards M1-like and M2-like states has been demonstrated^12-14^. In carp, we previously showed that macrophages assume an inflammatory phenotype in response to lipopolysaccharide (LPS) stimulation^9^ in the presence or absence of interferon-gamma (Ifn-γ)^15^. This phenotype is characterized by the production of NO (as in mice) and pro-inflammatory cytokines similar to mammalian M1 macrophages when stimulated with LPS alone or in combination with IFN-γ, or granulocyte macrophage-colony stimulating factor (GM-CSF)^16^. At the other end of the spectrum, cyprinid macrophages adopt an anti-inflammatory phenotype characterized by elevated arginase activity when stimulated with Il-4/13^17,18^ or cAMP^9,17^. This phenotype is similar to mammalian M2 macrophages polarized by macrophage-colony stimulating factor (M-CSF), interleukin-4 (IL-4), interleukin 13 (IL-13) or extracellular cAMP^19^, which show comparable increased production of anti-inflammatory cytokines^16^. In vivo, macrophage polarization has been studied using *tnfα*/*mpeg1*^20^ double transgenic zebrafish and preliminary findings show differences in expression of pro- and anti-inflammatory markers in *tnfα*^*+*^ and *tnfα*^*-*^ macrophages. In addition, some work towards a full transcriptional phenotype has been undertaken in *tnfα*/*mpeg1* double transgenic zebrafish^21^ and other teleosts^22,23^. Taken together, these studies provide the foundation to understand macrophage polarization in fish. However, a comprehensive transcriptomic analysis using known modulators of M1 and M2 polarization, associated with phenotypic validation through robust functional assays, is still lacking.

In this study, we used our well-established in vitro carp macrophage model and combined the phenotypically validated M1 and M2 macrophages with an unbiased transcriptome analysis to elucidate the transcriptional profile of M1 and M2 macrophages in a lower vertebrate. As such, we started with the functionally opposite ends of the macrophage spectrum, M1 and M2 extremes, which serve as a stable framework to determine evolutionary conserved polarization profiles. This allows us to comparatively study macrophage polarization across vertebrates and to identify a comprehensive set of genes that can be used as potential markers across species. In doing so, we provide insight into the conservation of macrophage polarization beyond mammals.

## Results

### Polarized macrophages show differences in morphology and in function

**Figure 1:**
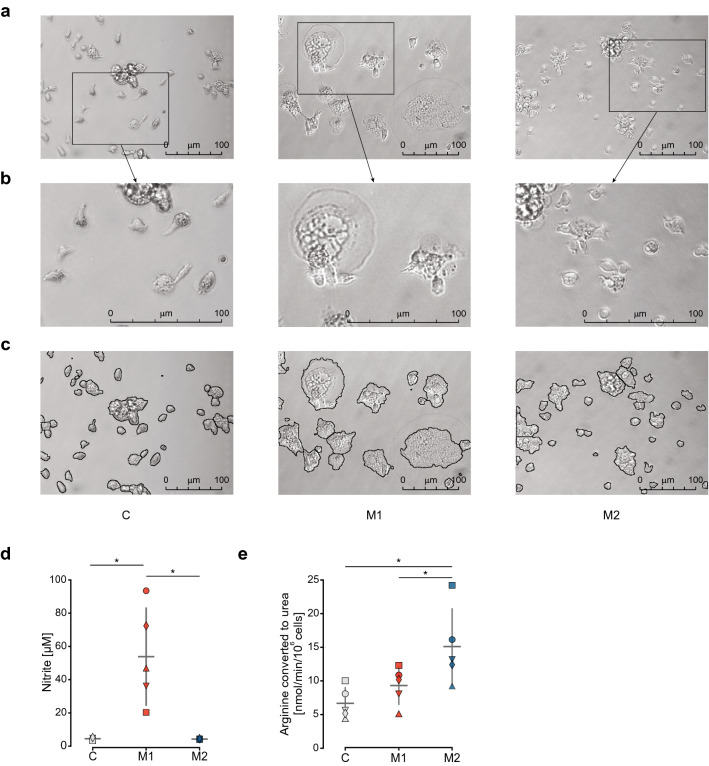
Polarized carp macrophages display different functional phenotypes. Phenotypical differences in carp macrophages either polarized for 24 h with 30 µg/ml LPS (M1) or 0.5 µg/ml cAMP (M2), or kept as unstimulated control (C) macrophages. (**a**) Representative images of macrophages from one individual, showing morphological differences. (**b**) Enlargement of areas indicated with boxes in panel a. (**c**) Tracing of cell edges with ImageJ accentuating morphological differences. (**d**) Nitric oxide production of control (grey), M1 (red) and M2 (blue) treated macrophages measured as nitrite concentration in culture supernatants after 24 h. Symbols indicate individual fish. (**e**) Arginase activity of control (grey), M1 (red) and M2 (blue) stimulated macrophages measured in cell lysates as conversion of L-arginine to urea by arginase in nmol/min/10^6^ cells. Symbols indicate individual fish. Data are the mean and standard deviation of *n* = 5 individual fish (d,e). Data were analyzed using a repeated measures ANOVA with Tukey post-hoc tests for NO and arginase assays (d,e). Differences were considered significant when *p* < 0.05 (*). In cases where sphericity was violated (e), the Geisser-Greenhouse correction was applied.

We studied the phenotype of stimulated carp macrophages to confirm their polarization state prior to transcriptome analysis. When macrophages were stimulated with LPS or cAMP, to obtain M1 or M2 polarization states respectively, we observed a change in morphology. During the polarization period of 24 h, when compared to unstimulated cells from the same individual, M1 macrophages adhered to the culture surface and assumed flattened, irregular shapes with multiple membrane protrusions, while M2 macrophages retained a more compact and rounded shape with only few protrusions (Fig. 1a–c). In addition, M1 macrophages formed a higher number of large, multinuclear cells (sometimes referred to as giant cells) compared to M2 macrophages or unstimulated controls. This difference in morphology was mirrored by clear differences in functional phenotypes. Analysis of two canonical macrophage functions, NO production (Fig. 1d) and arginase activity (Fig. 1e), showed clear differences between M1 and M2 macrophages. M1 macrophages showed a significantly higher cumulative NO production over 24 h than M2 and unstimulated controls, reflecting a pro-inflammatory phenotype. In contrast, M2 macrophages did not produce any NO and showed a significantly higher intracellular arginase activity than M1 and unstimulated macrophages. The clear functional difference in NO production and arginase activity indicates the capacity of teleost macrophages to assume M1 and M2 phenotypes similar to those in mammals.

### M1 and M2 carp macrophages display distinct gene expression profiles

**Figure 2:**
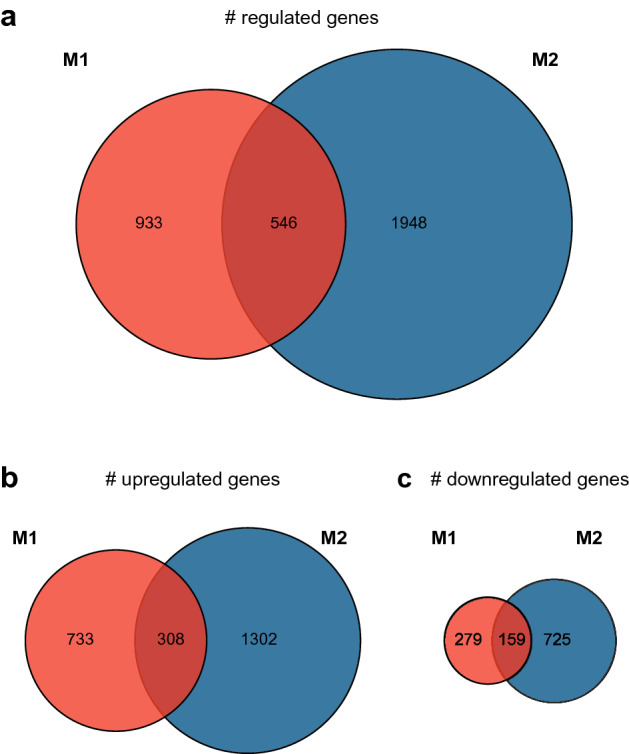
M1 and M2 carp macrophages show distinct transcriptional profiles. Proportional Venn diagrams depicting transcriptional changes of carp macrophages polarized for 6 h with 30 µg/ml LPS (M1, red) or 0.5 µg/ml cAMP (M2, blue) compared to unpolarized control macrophages. The total number of significantly (*p*_adjusted_ < 0.05) regulated genes (**a**) is further specified to show the number of upregulated (**b**) and downregulated (**c**) genes. Data are of n = 3 fish.

After observing clear morphological and functional differences between M1 and M2 carp macrophages, we examined their transcriptome at an earlier timepoint (6 h) to explore the differences in expression profiles preceding the observed changes in morphology, NO production and arginase activity. DESeq2 analysis resulted in 3396 significantly regulated genes in M1 macrophages and 6142 significantly regulated genes in M2 macrophages, compared to unstimulated control macrophages. Of those significantly regulated genes, expression of 1479 (M1) and 2494 (M2) genes was at least twofold increased or twofold decreased (log2 fold change > 1 or log2 fold change < − 1) and was thus defined as differentially expressed. Comparison of these genes (Fig. 2a) showed clearly distinct expression profiles since, besides the 546 genes regulated in both M1 and M2 macrophages, the majority was regulated only in M1 (63%, 933 genes) or only in M2 (72%, 1948 genes). Overall, more genes were up- than down-regulated, over 70% of which was upregulated either only in M1- or only in M2 macrophages, while 308 genes were upregulated in either group, representing less than 30% overlap (Fig. 2b). Similarly, at least 64% of downregulated genes are specific to either M1 or M2 macrophages, while only 36% or less overlapped (Fig. 2c). Taken together, these results show two distinct transcriptional profiles for polarized M1 and M2 carp macrophages.

### Distinct transcriptional profiles feature conserved pro-and anti-inflammatory genes

An analysis of GO terms associated with the full set of up- or downregulated genes (log2 fold change > 1 or log2 fold change < − 1) revealed enriched pathways that could be considered typical for M1 or M2 macrophages (Supplementary Table 1). For example, more specific GO terms enriched in M1 macrophages include ‘defense response’, ‘response to bacterium’ and ‘prostaglandin-endoperoxide synthase activity’. More specific GO terms enriched in M2 macrophages include ‘angiogenesis’, ‘transforming growth factor beta receptor signaling pathway’ and ‘3′,5′-cyclic-AMP phosphodiesterase activity’. Although informative, these pathway analyses exclude genes without GO identifiers and multiple paralogs in (duplicated) genomes of tetraploid fish such as carp^24^.

**Table 1:** Transcriptional phenotype of carp M1 macrophages shows high increases in inflammatory mediators and M1 markers.

Gene	Gene description	Gene ID cypCar	Log2 FC	Main function	RPKM C	RPKM M1
*il12p35*	Interleukin 12 subunit alpha (p35)	00024698–00024699	8.8 7.2	P35 subunit of the pro-inflammatory cytokine Il-12. Involved in the activation of Th1 and NK cells^25^	0.1 0.4	56.0 59.4
*il1β*	Interleukin 1 beta	00043439–00043440	7.6 7.5	Pro-inflammatory cytokine. Mediator of various cellular activities including proliferation, differentiation and apoptosis^26^	72.0 74.0	12832.1 11482.7
*steap4*	Six-transmembrane epithelial antigen of prostate 4	00042005	7.0	Metalloreductase involved in the transfer of ions from Fe_3_^+^ and Cu_2_^+^ to NAD and plays a role in cellular homeostasis during inflammation. Increased Steap4 may reduce circulating iron available for parasites^27^	4.6	405.9
*agrn*	Agrin	00029572	7.0	Extracellular-matrix protein involved in monocyte/macrophage survival, cytoskeleton formation and phagocytosis^28^	32.7	2569.8
*saa*	Serum amyloid A protein	0003733300036204	6.3 5.3	Acute phase protein, chemotactic to phagocytes and induces transcription of several pro-inflammatory cytokines^29,30^	2.8 77.6	183.8 1968.3
*ptgs2a or cox2*	Prostaglandin-endoperoxide synthase 2a	00026925	5.7	Also known as Cox-2. Increased expression in human M1 macrophages^31,32^	8.7	319.8
*olfm4*	Olfactomedin-4-like	00047183	5.4	Extracellular glycoprotein indicated in myeloid-specific differentiation and neutrophil inflammation^33-35^	1.8	69.5
*lacc1*	Laccase-domain containing protein 1	00009189	5.3	Promotes fatty-acid oxidation, inflammasome activation, mitochondrial and NADPH-oxidase-dependent reactive oxygen species production and bactericidal activity of macrophages^36^	4.3	137.2
*nos2b*	Nitric oxide synthase 2b	00004424 00024539	5.3 5.2	Production of antimicrobial nitric oxide. Has functioned as M1 marker since macrophage polarization was described^1,37^	10.1 43.6	355.4 1427.6
*mecr*	Mitochondrial Enoyl-[acyl-carrier-protein] reductase	00002503–00002502	5.1 5.0	Protein involved in mitochondrial fatty acid synthesis. Increased upon *Salmonella enteritidis* infection in chicken macrophages^38^	13.6 24.9	340.7 627.8
*il6*	Interleukin-6	00035927	5.0	Pro- and anti-inflammatory cytokine produced by macrophages in response to PRR activation^16,39^	30.2	864.5
*tdh*	L-threonine dehydrogenase	00008269	4.6	Converts L-theonine into glycine. Glycine modulates macrophage activity, plays a role in preventing pyroptosis and shows cytoprotective effects under hypoxia and oxidant injury^40,41^	16.3	356.7
*acod1 or irg1*	Aconitate decarboxylase 1 / Immune responsive gene 1	00007903 00026281	4.6 4.5	Catalyzes production of itaconate. High expression in mammalian M1 macrophages contributes to metabolic reprogramming^42,43^	71.5 6.4	1404.8 121.8
*cygb1*	Cytoglobin 1	00046202	4.3	Oxygen-carrying globin, expressed in macrophages and increased during oxidative stress. Protection mechanism against oxidative stress^44,45^	3.0	56.4
*cxcl13*	C-X-C motif chemokine ligand 13	00002926	4.0	B-cell chemoattractant. Upregulated in human M1 macrophages^31^	8.6	96.4
*cxcl8l1*	C-X-C motif chemokine ligand 8 like 1	00016657	4.0	Previously known as Cxca, Teleost specific Cxcl8-like cytokine^46^. Recruits neutrophils through CXCR2^47,48^	230.4	2984.1
*tymp*	Thymidine phosphorylase	00038018 00038017	3.7 3.6	Also known as platelet-derived endothelial-cell growth factor. Angiogenic factor expressed in macrophages^49^	24.8 22.4	215.9 204.8
*si:ch1073-67j19.1*	Unknown protein	00039673	3.6		314.3	3261.0
*tnfrsf11b*	tumor necrosis factor receptor superfamily, member 11b	00045494	3.6	Also known as osteoprotegerin, a secreted RANKL decoy receptor. Correlates with inos + macrophages antiapoptotic signal in DC leading to increased T-cell activation^50^	24.6	247.3
*shc2*	SHC transforming protein 2	00020157	3.5	Mediator of certain growth-factor signaling cascades. Implicated in cellular proliferation, differentiation, survival and migration^51^	4.9	50.3

Genes most upregulated (top 20) in M1 macrophages polarized with 30 µg/ml LPS for 6 h in descending order of fold change gene expression. Genes were included only when all of the following criteria were met: *p*_adjusted_ < 0.05 and average reads per kilobasepair per million reads (RPKM) > 50 in stimulated or control samples. The 20 most highly upregulated distinct genes were depicted with the gene abbreviation (Gene), gene description, gene identifier (Gene ID cypCar), log2 fold change compared to unstimulated control macrophages (Log2FC), short description of their main function (in macrophages if possible) and average RPKM in control (C) and LPS polarized macrophages. Multiple cypCar IDs per gene were included only if RPKM of both paralogs fell within the top 20 most upregulated genes. Each cypCar gene ID represents an individual gene sequence unless combined by a dash (–), indicating a possible mis-annotation of a single gene as two separate genes. Data are of *n* = 3 fish.

^1^Mills et al., 2000, ^16^Mantovani et al., 2004,^25^Wojno et al. 2019, ^26^Mantovani et al., 2019, ^27^Scarl et al., 2017, ^28^Mazzon et al., 2012, ^29^Badolato et al., 1994, ^30^He et al., 2009, ^31^Martinez et al., 2006, ^32^Jablonski et al., 2015, ^33^Zhang 2002, ^34^Clemmensen et al., 2012, ^35^Alder et al.,2018, ^36^Lahiri et al., 2017, ^37^Nathan et al., 1991, ^38^Sekelova et al., 2017, ^39^Beyer et al., 2012, ^40^Carmans et al., 2010, ^41^Loomis et al., 2019, ^42^Van den Bossche et al., 2017, ^43^O’Neill et al., 2019, ^44^Li et al., 2007, ^45^Oleksiewicz et al., 2011, ^46^Abdelkhalek et al., 2009, ^47^Deng et al., 2013, ^48^de Oliveira et al., 2013, ^49^Fox et al., 1995, ^50^Walsh and Choi, 2014, ^51^Ahmed and Prigent, 2017.

To further define the expression profiles of M1 and M2 polarized macrophages, we focused the analysis to those genes that are not only differentially but also substantially expressed in either polarized or unstimulated control macrophages (full lists in Supplementary Table 2). We define genes substantially expressed when RPKM > 50 in either polarized or unstimulated control macrophages and for upregulated genes a log2 fold change > 1). In our dataset, these represent approximately 7–10% of all significantly regulated genes. Here we only highlighted the genes that show the highest (top 20) fold change in expression in M1 (Table 1) or M2 (Table 2) macrophages compared to unstimulated controls. Only four out of the 20 most upregulated genes show overlap between M1 and M2 macrophages. These are genes involved in general cellular (activation) processes such as cytoskeleton formation (*agrn*), growth-factor signaling (*shc2*) and amino-acid metabolism (*tdh*). Although we observed the canonical pro-inflammatory cytokine *il1β* in both groups, expression was approximately 30 times higher in M1 than in M2 macrophages, which indicates it acts primarily as a pro-inflammatory M1 gene.

**Table 2:** Transcriptional phenotype of carp M2 macrophages shows high increases in mediators of tissue regeneration and M2 markers.

Gene	Gene description	Gene ID cypCar	Log2FC	Main function	RPKM C	RPKM M2
*cyr61l1*	Cysteine-rich angiogenic inducer 61 protein-like protein 1	00001309	9.4	Also known as *cnn1*. Extracellular matrix protein involved in angiogenesis and regulation of matrix remodeling in cutaneous wound healing. Drives an anti-inflammatory transcriptional profile^52,53^	0.2	103.8
*timp2b*	Tissue inhibitor of metallo-proteinase 2b	00030755 00034223	8.0 4.2	Inhibits metalloproteinases and is involved in extracellular matrix remodeling. Decreased in M1 macrophages and increased in M2^54^	31.1 194.3	6476.0 2787.4
*tgm2b*	Transglutaminase 2b protein	00034483–00030329 00041907	7.4 6.9 5.0	Ca2 + -dependent cross-linking enzyme important in apoptotic cell clearance by phagocytosis and regulation of pro-inflammatory cytokine production. Conserved M2 marker in human and murine M2 macrophages^55-57^	6.0 6.4 1.7	784.6 698.7 68.2
*ramp2*	Receptor activity modifying protein 2	00022158	6.4	Involved in glycosylation and transportation of the adrenomedullin receptor to the cell surface^58^. Adrenomedullin is associated with angiogenesis and M2 macrophage phenotypes, especially in the context of cancer^59,60^	1.8	100.9
*dfna5a* or *gsdmea*	Deafness autosomal dominant 5a/ Gasdermin Ea	00035581	5.1	Considered the functional homologue in zebrafish of human gasdermin E. Although generally an effector of pyroptosis, that role has been recently questioned specifically in macrophages^61-63^	12.8	421.8
*arg2*	Arginase 2	00034978	4.8	Arginase 1 is the canonical M2 marker in murine M2 macrophages. In human M2 macrophages dependent on the study^1,64^	19.9	445.2
*agrn*	Agrin	00029572	4.3	Extracellular-matrix protein involved in monocyte/macrophage survival, cytoskeleton formation and phagocytosis^28^	11.2	153.8
*pde4bb*	Phosphodiësterase 4b	00024882 00020192	4.3 3.6	Degrades second messenger cAMP, promoting pro- and regulating anti-inflammatory effects^65-67^	17.4 25.4	284.8 266.8
*vegfaa*	Vascular endothelial growth factor Aa	00013154	4.3	Signaling protein involved in angiogenesis and tissue generation. Upregulated in M2 macrophages^68,69^	4.9	77.9
*csrnp1a*	Cysteine-serine-rich nuclear protein 1a	00015701	4.2	Transcriptional activator involved in Wnt-signaling and involved in primitive hematopoiesis in zebrafish^70^. Upregulated in macrophages of different origins with multiple stimuli including murine BMDM with Il-13^71^or LPS^72^	7.5	117.1
*il1β*	Interleukin 1 beta	00043439 00043440	4.2 4.0	Pro-inflammatory cytokine. Mediator of various cellular activities including proliferation, differentiation and apoptosis^26^	46.1 42.2	945.4 771.8
*hbegfb*	Heparin-binding EGF-like growth factor b	00014699	4.2	Soluble and membrane bound forms. Growth factor in early stages of wound healing. Promotes dermal repair, angiogenesis and is expressed by anti-inflammatory macrophages^73,74^	7.8	129.2
*angptl4*	Angiopoietin-like 4	00035942 00049924	4.1 3.9	Downregulated by TLR-stimulation in macrophages, prevents the formation of lipid-laden giant cells^75^ and associated with anti-inflammatory macrophages^76,77^	20.1 20.6	260.6 244.3
*steap4*	Six-transmembrane epithelial antigen of prostate 4	00042005	3.8	Metalloreductase involved in the transfer of ions from Fe_3_^+^ and Cu_2_^+^ to NAD and plays a role in cellular homeostasis during inflammation. Increased Steap4 may reduce circulating iron available for parasites^27^	6.8	100.6
*ppap2b* or *plpp3*	Phosphatidic acid phosphatase type 2B/ Phospholipid phosphatase 3	00003642 00045370	3.8 3.7	Lysophosphatidic acid (LPA) inhibitor. Induced by VEGF and involved in angiogenesis^78^ and favors anti-inflammatory phenotypes^79,80^	8.7 20.8	100.2 222.4
*tdh*	L-threonine dehydrogenase	00008269	3.8	Converts L-theonine into glycine. Glycine modulates macrophage activity, plays a role in preventing pyroptosis and shows cytoprotective effects under hypoxia and oxidant injury^40,41^	21.6	286.4
*tgm1l1*	Transglutaminase 1-like 1	00018981	3.8	Tgm1 is a cross-linking enzyme involved in tissue regeneration. Upregulated in macrophages in response to M-CSF^57^	45.6	509.5
*crema*	cAMP-responsive element modulator a	00009477	3.7	Involved in cAMP signaling. Binds cAMP response element and different splice variants act as both enhancers and repressors of transcription^81^	10.0	108.6
*cremb*	cAMP-responsive element modulator b	00033214	3.6	Involved in cAMP signaling. Binds cAMP response element and different splice variants act as both enhancers and repressors of transcription^81^	15.6	164.5
*shc2*	SHC-transforming protein 2	00020157	3.6	Mediator of certain growth-factor signaling cascades. Implicated in cellular proliferation, differentiation, survival and migration^51^	5.7	58.8

Genes most upregulated (top 20) in M2 macrophages polarized with 0.5 mg/ml cAMP for 6 h in descending order of fold change gene expression. Genes were included only when all of the following criteria were met: *p*_adjusted_ < 0.05 and average reads per kilobasepair per million reads (RPKM) > 50) in stimulated or control samples. The 20 most highly upregulated distinct genes were depicted with the gene abbreviation (Gene), gene description, gene identifier (Gene ID cypCar), log2 fold change in compared to unstimulated control macrophages (Log2FC), short description of their main function and average RPKM in control (C) and cAMP polarized macrophages. Multiple cypCar IDs per gene were included only if RPKM of both paralogs fell within the top 20 most upregulated genes. Each cypCar gene ID represents an individual gene sequence unless combined by a dash (–), indicating a possible mis-annotation of a single gene as two separate genes. Data are of *n* = 3 fish.

^1^Mills et al., 2000, ^28^Mazzon et al., 2012, ^52^Chen et al., 2001, ^53^Chen and Lau, 2009, ^54^Orecchioni et al., 2019, ^55^Martinez and Gordon, 2014, ^56^Nadella et al., 2015, ^57^Sun and Kaartinen., 2018, ^58^McLatchie et al., 1998, ^59^Chen et al., 2011, ^60^Pang et al., 2013, ^61^Rogers et al., 2017, ^62^Chen et al., 2019, ^63^Broz et al., 2019, ^64^Munder et al., 1999, ^65^Jin et al., 2005, ^66^Hertz et al., 2009, ^67^Yang et al., 2017, ^68^Stockmann et al., 2011, ^69^Roszer et al., 2015, ^70^Espina et al., 2013, ^71^Das et al., 2018, ^72^Eichelbaum and Krijgsveld, 2014, ^26^Mantovani et al., 2019, ^73^Shirakata et al., 2005, ^74^Edwards et al., 2009, ^75^Oteng et al., 2019, ^76^Feingold et al., 2009, ^77^Cho et al., 2019, ^27^Scarl et al., 2017, ^78^Wary and Humtsoe 2005, ^79^Gustafsson et al., 2008, ^80^Panchatcharam et al., 2014, ^40^Carmans et al., 2010, ^41^Loomis et al., 2019, ^81^Della Fazia et al., 1997, ^51^Ahmed and Prigent, 2017.

Next to *il1β,* many other genes among the 20 most upregulated genes in M1 macrophages agree with the prototypical M1 profile. This includes pro-inflammatory cytokines *il12p35* and *il6*, the acute phase protein *serum amyloid a* (*saa*) and genes contributing to or protecting from oxidative stress (*nos2b, irg1, lacc1* and *cygb1*). These genes do not only functionally suit an inflammatory profile, but many of these genes have also been previously linked to human or murine M1 polarized macrophages.

Many of the 20 most upregulated genes in M2 macrophages (Table 2) agree with the prototypical M2 profile as described in mammals. Some of these have even been proposed as M2 markers, such as *cyr61(l1), timp2(b)* and *tgm2(b)*. Other genes, such as *vegfa(a)* and *csnrp1(a)*, have been linked to M2 profiles via transcriptional studies in mammals or can be linked to M2 macrophages on a functional level. For example, some genes are involved in angiogenesis and wound healing (*hegf(b)*, *tgm1l*, *vegfa(a)*, *cyr61(l1)*), while others facilitate either transcription (*crem (a* and *b*) or the presence of M2 associated receptors (*ramp2*). Overall, the transcriptional M1 and M2 profiles studied here are distinct from each other and show upregulation of genes associated with M1 and M2 transcriptional profiles in mammals.

### Transcriptional profiles of M1 and M2 macrophages are enhanced by T-helper cell associated cytokines

Mammalian IFN-γ is known to activate pro-inflammatory (M1) functions of macrophages, especially when macrophages are co-stimulated with potent microbial stimuli such as LPS. Mammalian IL-4 is known to activate anti-inflammatory (M2) functions of macrophages, also when administered without co-stimuli. We studied enhancement of macrophage function by carp Ifn-γ in a co-stimulation experiment with LPS by comparing transcription profiles with, and without the presence of carp Ifn-γ. The majority of differentially expressed genes overlapped between both groups, representing almost 90% of genes in LPS-only stimulated macrophages and almost 70% in LPS + Ifn-γ stimulated-macrophages (Supplementary Fig. 1a). Similar percentages were found for both up- and downregulated genes (Supplementary Fig. 1b and c). Many of the overlapping and most-upregulated genes were even higher expressed in macrophages stimulated with the combination of LPS and Ifn-γ (Supplementary Table 3), suggesting that Ifn-γ enhances the gene profile already induced by LPS alone. Indeed, all genes listed in the top 20 except *olfm4* and *mecr*, showed higher fold-changes in co-stimulated macrophages. Also, several genes of interest upregulated in macrophages stimulated with LPS alone but below the arbitrary threshold of 50 RPKM, such as *mhc2dbb*, *mpeg1.2*, and *tmem238,* were now among the top 20 upregulated genes. Together, these results indicate that Ifn-γ can enhance the pro-inflammatory profile induced by LPS alone while retaining the conserved M1-like marker profile.

We also set out to determine the effect of a carp Il-4/13 paralog on carp macrophages by comparing the induced transcription profile with the one of unstimulated macrophages (to ultimately compare with cAMP-stimulated macrophages) but could not detect consistent transcriptional changes different from those in unstimulated control macrophages. The unresponsiveness of carp macrophages to Il-4/13 was not due to lack of bioactivity of the recombinant Il-4/13b1 which was confirmed by a downregulation of pro-inflammatory responses induced in mid-kidney leukocytes (Supplementary Fig. 2); a result similar to what has been observed in grass carp^18^. The unresponsiveness of carp macrophages to Il-4/13 was likely not due to lack of an Il-4/13 sensitive receptor complex on unstimulated macrophages, because the presence of receptors and transcription factors likely involved in Il-4-induced signaling could be identified in unstimulated carp macrophages. These were identified based on known receptor complexes in mammals and on published homologs in zebrafish^82^ and grass carp^83^ and included a putative Il-4R*α *chain, two putative paralogs of the IL-13Rα1 chain, two putative paralogs of the IL-13Rα2 chain and three putative paralogs of the common gamma chain γc. All receptors were expressed at substantial levels of 30–600 RPKM (Supplementary Table 4) in unstimulated (control) macrophages. In addition, we could confirm expression of both *stat6* and *stat3* downstream transcription factors at values of 20–100 RKPM (Supplementary Table 4) in unstimulated (control) macrophages. These results show that the main signaling components of the Il-4 pathway are present and expressed in carp macrophages. Overall, our results indicate that further research into the function of all Il-4/13 paralogs carp needs to be performed before a statement can be made on the ability of carp Il-4/13 to induce an anti-inflammatory (M2-like) profile.

### Transcriptional analysis reveals candidate markers to discriminate between M1- and M2-macrophages

**Figure 3:**
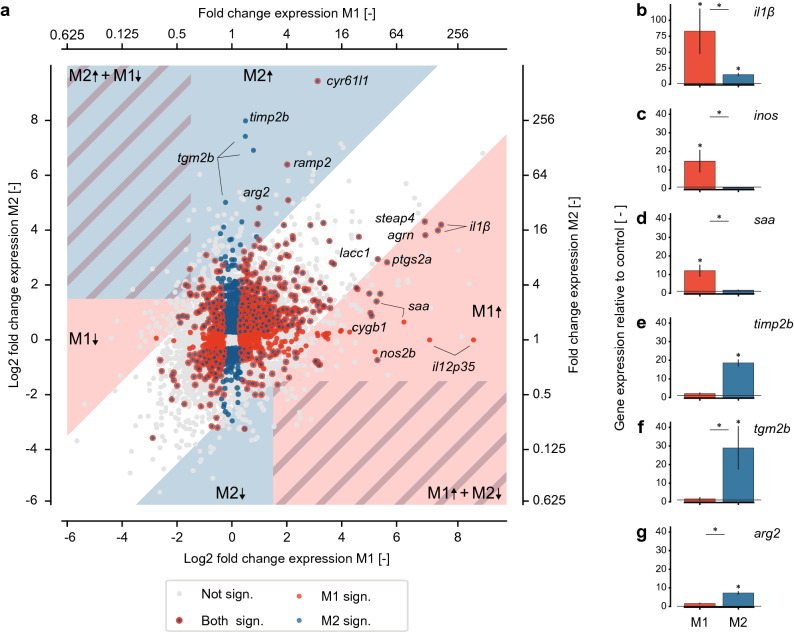
Graphical representation of transcriptional data reveals candidate markers for M1 and M2 macrophages in carp. **(a**) Graphical representation of transcriptional profiles of carp macrophages polarized for 6 h with 30 µg/ml LPS (M1) or 0.5 µg/ml cAMP (M2) compared to unpolarized control macrophages. Dots represent genes with an average number of reads > 50 reads per kilobasepair per million reads (RPKM) in either stimulated or unstimulated control macrophages. Grey dots indicate genes that are not significantly regulated in either M1 or M2 macrophages (*p*_adjusted_ > 0.05). Red dots indicate genes that are significantly regulated in M1 macrophages (*p*_adjusted_ < 0.05). Blue dots indicate genes that are significantly regulated in M2 macrophages (*p*_adjusted_ < 0.05). Blue dots with red edges indicate genes that are significantly regulated in M1 and M2 macrophages (*p* < 0.05). Position on the *x*-axis represents the average log2 fold change (bottom axis) or fold change (top axis) of LPS stimulated macrophages compared to unstimulated controls. Position on the *y*-axis displays the average log2 fold change (left axis) or fold change (right axis) of cAMP stimulated macrophages compared to unstimulated controls. Dots within the translucent area represent potential marker genes that change at least 1.5 log2 fold from unstimulated controls (log2 fold < − 1.5 or log twofold > 1.5). Dots in the red translucent area represent genes that are at least 2.5 log2 fold higher or lower expressed in M1 macrophages then M2 macrophages. Dots within the blue translucent area represent genes that are at least 2.5 log2 fold higher or lower expressed in M2 macrophages then M1 macrophages. Dots in the striped translucent area represent potential marker genes that inversely regulated in M1 and M2 macrophages. Labels with arrows indicate whether genes are up- or downregulated. Examples of good potential marker genes are labeled. Additional marker candidates are included as supplementary data (Supplementary table 5). Data are of *n* = 3 fish. Real-time qPCR analysis of gene expression of *il1β* (**b**), *inos* (*nos2b*) (**c**), *saa* (**d**), *timp2b* (**e**), *tgm2b* (**f**) and *arg2* (**g**) using common primers for paralog sequences confirms these genes as appropriate markers for polarized macrophages. Gene expression was normalized to the *s11* protein of the *40 s* subunit as a reference gene and shown as the fold change relative to the unstimulated controls (line at *y* = 0). Data are the mean and standard deviation of *n* = 4. Data were analyzed using a repeated measures ANOVA with Dunnett’s T3 post-hoc tests for unequal variances or the Kruskal–Wallis test in case normality was violated. Differences were considered significant when *p* < 0.05 (*).

To be able to read-out polarized macrophage responses in future studies in teleost fish, we propose a set of appropriate candidate markers for M1 and M2 macrophages identified in this study for carp. We identified as appropriate candidate markers those genes that are not only significantly regulated or only highly regulated but also sufficiently specific for either M1, or M2 macrophages. First, we determined for all regulated genes their relative expression in M1 and M2 macrophages compared to unstimulated controls (Fig. 3a). We then determined which genes were significantly regulated only in M1 (red dots), only in M2 (blue dots) or regulated in both M1 and M2 macrophages (blue dots with red edge). We included as appropriate candidate markers those genes significantly up- or downregulated in only one group. Additionally, we included those genes significantly up- or downregulated in both groups, as long as the differences in fold changes are large enough to distinguish between M1 and M2 macrophages. For example, *il-1β* is significantly upregulated in both M1 and M2 macrophages, but with a fold change of 187.5 (7.55 log2 fold ) in M1 sufficiently different from the 17 fold change (4.1 log2 fold) in M2 to keep *il-1β* as an informative marker gene for M1 macrophages. With this in mind, we identified as suitable candidate marker genes those that fit the two following criteria: (1) an expression at least 1.5 log2 fold up- or downregulated compared to unstimulated controls and (2) a ratio of gene expression between M1 and M2 of at least 2.5 log2 fold. The latter means that a potential M1 marker is at least 5.7-fold higher expressed in M1 than in M2 and vice versa.

According to above-described criteria, all genes within the shaded areas of Fig. 3a are potential marker genes for M1 (red shade) or M2 (blue shade) macrophages (specified in Supplementary table 5). Of high interest, genes within striped areas represent genes that fit these criteria for both M1 and M2 subsets and are oppositely regulated in M1 versus M2. These genes are therefore among the most specific marker genes (specified in Supplementary table 5). For six potential candidate markers for M1 (*il1β, nos2b* and *saa*) and M2 (*timp2b*, *tgm2b* and *arg2*) macrophages we validated their suitability for detection by real-time qPCR. We confirmed a significant increase in *il1β*, *nos2b* and *saa* expression in M1 but not in M2 macrophages (Fig. 3b–d). Likewise, we confirmed a significant increase in *timp2b*, *tgm2b* and *arg2* in M2 macrophages but not in M1 macrophages (Fig. 3e-g). This suggests these markers, among others, are suitable for gene expression studies on polarized macrophages populations. It also highlights these genes as valuable targets for additional approaches such as the development of specific antibodies or the generation of zebrafish transgenic reporter lines which would both allow to study macrophage polarization at the cellular level, if not in vivo.

## Discussion

In this study we performed a comprehensive analysis of the transcriptional profile of M1- and M2-like polarized macrophages from a teleost fish and compared the genes highest expressed with those known for mammalian M1 and M2 counterparts. We used LPS and cAMP as main stimuli and first confirmed M1- and M2-like functional phenotypes of macrophages from common carp, which were subsequently used for RNA sequencing. The resulting transcriptional profiles of carp macrophages show a high degree of conservation with those of polarized macrophages as we know them today from humans and mice. These profiles provide an unbiased and solid framework to not only confirm previously used markers but select additional markers of polarized macrophage responses in a non-mammalian species.

The classical approach of using cytokine stimuli to polarize mammalian macrophages may not necessarily be directly applicable, nor needed, for studies on fish macrophages. Macrophages of mice and humans have traditionally been polarized with microbial stimuli such as LPS combined with cytokines associated with Th1 (IFN-γ) for M1 macrophages and have traditionally been polarized with cytokines associated with Th2 responses (IL-4) for M2^84,85^. Furthermore, addition of the growth factors GM-CSF or M-CSF help to induce polarization towards M1 or M2 phenotypes, respectively^86^. For studies on fish macrophages it is not always possible nor evident to copy these exact experimental set-ups. In fish, the degree of functional conservation of the cytokines IFN-γ and IL-4 remains subject of discussion^87,88^, with evidence of their ability to induce polarized T cell-mediated responses being stronger for IFN-γ^89^ than for IL-4^90,91^. Moreover, although the presence of M-CSF has been studied at expression level^92^ and effects on macrophage proliferation have been reported^93^, evidence of the presence of GM-CSF in fish genomes remains elusive^94,95^. It has been shown that macrophages of goldfish and carp can be stimulated with LPS^9^ alone to induce M1-like phenotypes producing nitric oxide, or with cAMP^9,17^ to induce M2-like phenotypes displaying arginase activity. Indeed, it is plausible that the initial trigger for macrophage polarization into M1 or M2 could rely primarily on sensing microbial/parasite infection or other innate danger signals, without a required presence of T-cell derived cytokines. This reverts the idea of the dichotomous Th1 and Th2 driving forces by suggesting that polarized innate immune responses could drive polarized adaptive responses, a concept described as ‘the macrophages first’ hypothesis^5,13^.

We primarily used innate immune stimuli (LPS, cAMP) to stimulate carp macrophages and determine subsequent polarized phenotypes with differences in morphology, function and transcriptional profiles. Importantly, the gene expression profiles of these polarized M1 and M2 macrophages of fish revealed upregulation of many genes also associated with the concurrent phenotypes in mammalian macrophages. Some of these genes have previously been associated with activated macrophages in varying fish species. For example, the chemokine *cxcl8l1* (otherwise known as *cxca*) was mentioned as *cxcl8a* and specifically expressed in LPS-stimulated macrophages of grass carp. We likewise noticed a 16-fold upregulation in carp M1 macrophages. The chemokine *ccl20a* was also mentioned as increased in LPS-stimulated macrophages in grass carp^23^ in the same study and was also substantially (8 log2 fold) increased as one *ccl20a* paralog in carp M1 macrophages, although not among the most highly expressed genes. Chemokine receptors *cxcr3* were mentioned as markers of M1 (*cxcr3.1*) and M2 (*cxcr3.2*) macrophages of grass carp, ayu and spotted green pufferfish^22^. We likewise noticed an upregulation of *cxcr3.3*, which is closely related to *cxcr3.1*, in carp M1 macrophages, but were unable to confirm upregulation of *cxcr3.2* in carp M2 macrophages. The pro-inflammatory cytokines *il1β* and *il6* were mentioned as highly expressed in *mpeg1*^+^ M1 (*tnf1*^+^) macrophage subsets of zebrafish, and the chemokine receptor *cxcr4b* and *alox5ap*, required for leukotriene synthesis, mentioned as highly expressed in M2 (*mpeg1*^+^/*tnf1*^-^) macrophage subsets^21^. We likewise noticed an increase of *il1β* and *il6* in carp M1 macrophages and of *cxcr4b* and *alox5ap* in M2 carp macrophages. Among other M1 markers, the chemokine *cxcl11* was mentioned as upregulated after mycobacterial infection in zebrafish larvae^21^. This typical M1 marker showed a strong decrease of expression in carp M2 macrophages. In summary, our data provides a combination of functional and comprehensive, unbiased transcriptional information on fish macrophages polarized towards both M1 and M2 polarization states. Our gene expression profiles on carp macrophages unite several observations of others on macrophages from different fish species. Our data not only indicate that the distinct gene expression profiles of carp macrophages are indeed distinct M1- and M2-like profiles but also highlight M1- and M2-specific gene transcription profiles show a striking conservation from teleost fish to mammals.

Our data suggest that carp macrophages could be polarized by innate damage and danger signals without the presence of T-cell derived cytokines and thus provide support to the ‘macrophages first’ point of view^13^. As mentioned before, for studies in fish it is not always evident to copy the common practice of co-stimulating macrophages with the cytokines IFN-γ and IL-4 to polarize into M1 or M2 states. Still, it remains of interest to study the effect of these cytokines on fish macrophages, primarily to investigate evolutionary conservation of cytokine function. Although co-stimulation of carp macrophages with recombinant carp Ifn-γ upregulated genes additional to those upregulated by LPS alone, the major effect of Ifn-γ was an amplification of the expression of the majority of the genes also upregulated by LPS stimulation alone. This hints at a certain degree of conservation of function for Ifn-γ with respect to macrophage activation. In contrast to observations in other teleost species^17,18^, we could detect no effect of carp Il-4/13b1 on macrophages, despite evident bioactivity of the recombinant protein. The presence in carp macrophages of a putative Il-4/13 sensitive receptor complex and the machinery for Il-4/13 signaling suggests there could be sub-functionalization in function or target^24^ between different Il-4/13 paralogs. Preliminary analysis of the common carp genome revealed genes encoding at least four different ll-4/13 paralogs, indicating the possibility for sub-functionalization. Alternatively, Il-4/13-like cytokines could function primarily as enhancer of pathways induced by, for example, microbial stimuli. Although we did not observe clear effects of Il-4/13b1 on the response of macrophages when added simultaneously with LPS (preliminary data not shown), it will be of great interest to study the effects of Il-4/13 paralogs on macrophages when combined with microbial stimuli added simultaneously or in sequence. Without studying the biological effect of all cytokine and receptor paralogs on fish macrophages either or not in combination with microbial stimuli, it is difficult to draw conclusions on the exact role and effect of these Il-4/13-like cytokines on fish macrophages.

Here, we provided a comprehensive list of candidate marker genes to help identify M1 and M2 fish macrophages. First, we could confirm using qPCR clear differences in gene expression between a number of well-known markers for M1 and M2 macrophages, including *il1β*, *inos* (*nos2b*), and *saa* for M1 macrophages and *timp2b*, *tgm2b* and *arg2* for M2 macrophages. These markers may be informative, but they are not always exclusive. For example, although *il1β* is much higher expressed in M1 macrophages, it is not absent in M2 macrophages. Moreover, expression of *tnfα*, which is commonly used to visualize inflammatory macrophages in transgenic zebrafish, is upregulated in carp M1 macrophages and downregulated in M2 macrophages as expected. However, differences in expression are small compared to other genes and it is readily detectable in M1, M2 and control macrophages (15–180 RPKM). Such observations indicate that other candidate markers may be even more suitable because they are more specific for a particular polarization state. Such markers would be up- or downregulated compared to controls in a specific macrophage subset and either remain the same or show opposite regulation in the other subset. We therefore set stringent thresholds for regulation and differences between subsets to provide more selective lists of candidate marker genes. For M1 macrophages, interesting additional candidate markers could include *heat-shock protein 70* (*hsp70*), as many *hsp70* paralogs are upregulated in M1 macrophages while they are slightly downregulated in M2 macrophages. In mammals, HSP70 prevents NO-induced apoptosis in macrophages^96,97^, indicating its functional significance in inflammatory macrophages. Hsp70 has also been indicated in antiviral responses in grass carp^98^. Another interesting candidate is *irg1*, because upregulation of both paralogs is increased to a much higher extent in M1 compared to the upregulation in M2 macrophages and is involved in the metabolic phenotype of these macrophages^42,43^. Particularly interesting is *cxcl11*, as this traditional human M1 marker is not only a good M1 marker for carp macrophages, but has been indicated as M1 marker in zebrafish as well^21^. For M2 macrophages, interesting additional markers could include the *mannose receptor c type 1b* (*mrc1b*) genes which are upregulated in M2 but downregulated in M1 macrophages. Indeed, the mannose receptor has been described and used as a M2 marker for human and murine M2 macrophages^85,99^. Furthermore, *angiopoietin-like 4* (*angptl4*) appears consistently upregulated in M2 macrophages only and is associated with M2 macrophage polarization and tissue repair in mammals^77^.

For macrophages of mammalian species it is becoming clear that subtle differences in polarization states exist between similar but distinct stimuli, both in vitro and in vivo^2,39^. This is reflected by the expansion of the number of defined macrophage phenotypes in mammals and the increasing support for a spectrum view on macrophage polarization^2,5^. Similarly, we expect a spectrum of macrophage polarization states in fish and advocate the use of a comprehensive set of markers as opposed to a single gene to discriminate between polarization states. Here, we have studied the phenotypes of M1 and M2 extremes in fish macrophages and proposed such markers to further characterize differences in macrophage polarization by using an approach which closely resembles the in vitro studies on bone marrow derived macrophages in mammals. Mammalian M2 macrophages have been divided into M2a (IL-4/IL-13), M2b (co-activated with immune complexes/apoptotic cells) and M2c (IL-10, TGF-β or glucocorticoid hormones) primarily based on their in vitro stimulus, and their resulting functions range from inducing type II immunity (M2a) to regulation of inflammatory responses (M2c)^16,100^. Although many of the same stimuli have been identified in fish, it is premature to conclude similar M2 subsets would also appear in fish. Yet, next to the Il-4/13 paralogs discussed above, Il-10 shows anti-inflammatory effects on carp macrophages in vitro^101^, as does cortisol^102^. The degree of conservation of possible M2-like subsets in fish remains to be determined in more detail, possibly using an approach similar to ours. Finally, the debate continues on how well cytokine-dependent in vitro phenotypes reflect those developing in the complex environment in vivo^1,54^. This question that may be addressed by further ex vivo characterization of macrophages polarized during infection^10^, or by studying macrophage behavior in vivo. We argue that the candidate markers from carp could aid the development of new transgenic zebrafish targeting M1 and M2 macrophages^13^. Transgenic zebrafish, well known for the possibility to visualize and follow specific immune cells in vivo^103,104^ may be of great help tracing M1- and M2-like macrophages in real time.

Last but not least, steering innate immune responses could provide a valuable alternative to the use of antibiotics and could replace or at least help vaccination in the quest to sustainably improve fish health in aquaculture, a form of animal production which is rapidly becoming more important^11^. The development of simple read-out systems can be crucial to the development of targeted innate immune stimulants that are able to steer macrophages towards the polarization state that is most effective against the pathogen at hand. In this study, we provide both transcriptional profiles and potential markers which will contribute substantially to the development of new read-outs to determine polarization states of the innate immune system.

## Materials and methods

### Experimental animals

European common carp (*Cyprinus carpio carpio L.*) used in experiments were the 12 months old offspring of a cross between the R3 strain of Polish origin and the R8 strain of Hungarian origin^105^. Carp were bred and reared in the aquatic research facility of Wageningen University and Research at 23ºC in recirculating UV-treated water and fed pelleted dry food (Skretting, Nutreco) twice daily. All experiments were performed with the approval of the Animal Experiments Committee of Wageningen University and Research (Ethical Committee documentation number 2017.W-0034) in accordance with the guidelines and regulations.

### In vitro culture and polarization of carp macrophages

Head kidney-derived macrophages were obtained as described previously^9^. In short, total head kidney leukocytes were cultured for 6 days at 27 °C, at a density of 17.5 × 10^6^ cells/75 cm^2^ flask in complete NMGFL-15 medium (incomplete -NMGFL15 supplemented with 5% pooled carp serum (PCS) and 10% bovine calf serum (Invitrogen Life Technologies) with 100 U/ml of penicillin G, 100 µg/ml of streptomycin sulfate (Gibco) and 50 µg/ml Gentamycin (Sigma-Aldrich) to obtain macrophages.

To polarize, macrophages were harvested by gentle scraping after incubation on ice for 15 min. Cells were pelleted at 450×*g* for 10 min at 4ºC before resuspension in cRPMI + (RPMI 1640 culture medium with 25 mM HEPES and 2 mM L-glutamine, supplemented with L-glutamine (2 mM), penicillin G (100 U/ml), streptomycin sulfate (100 µg/ml, Gibco) and heat-inactivated PCS (1.5% v/v)). Depending on the assay, macrophages were polarized for 6 h or 24 h with 30 µg/ml LPS (*Escherichia coli*, L2880, Sigma-Aldrich) with or without 100 ng/ml recombinant Ifn-γ for M1 macrophages, or with 0.5 mg/ml dibutyryl cAMP (N^6^,2′-O-dibutyryladenosine 3′:5′-cyclic monophosphate sodium D0627, Sigma-Aldrich, referred to as cAMP) or 100 ng/ml recombinant Il-4/13b1 for M2 macrophages, or with an equal volume of medium as unstimulated controls. Cells were cultured at 27˚C in the presence of 5% CO_2_.

### Functional and morphological confirmation of macrophage polarization

NO production was determined in culture supernatants of polarized macrophages. In brief, 5 × 10^5^ macrophages per well were seeded in 96-wells plates (Corning) in 150 µl of cRPMI + . After polarization, NO production was determined as nitrite in 75 µl culture supernatant as described previously^106^.

Arginase activity was measured in cell lysates as the amount of urea produced by the conversion of L-arginine to urea by arginase and normalized using a ratio of the sample protein content compared to lysate of control cells. A total of 1.5 × 10^6^ cells polarized for 24 h in 450 µl cRPMI + , were lysed in 100 µl of 0.1% Triton X-100. Protein content of the samples was determined using the Bradford protein dye reagent (Bio-Rad) according to the manufacturer’s protocol. Arginase activity was measured in 25 µl lysate as described previously for 50 µl lysate^9^, but volumes were scaled down accordingly. Arginase activity was determined as the conversion of L-arginine to urea by arginase and expressed in nmol/min/10^6^ cells.

For brightfield microscope images, 5 × 10^4^ macrophages polarized for 24 h in 150 µl cRPMI + in 96-wells plates (Corning) were imaged using a DMi8 inverted digital microscope (Leica Microsystems), controlled by Leica LASX software (version 3.4.2.) and equipped with 40x (NA 0.6) and 20x (NA 0.4) long distance objectives (Leica Microsystems). Highlighting of cell-edges was performed with ImageJ according to the pipeline of Choudhry^107^ with a final addition of the Find Edges function.

### Recombinant cytokines

Recombinant carp interferon gamma 2 (Ifn-γ) was produced as described previously^15^. Protein analysis by SDS-PAGE (12% Tris–HCl, Bio-Rad) stained with GelCode Blue Stain Reagent (Thermo Scientific) revealed proteins were at least 95% pure and the chromogenic Limulus amebocyte lysate end-point test (Charles River Laboratories) showed that the residual endotoxin content was below detection limit (< 0.15 EU).

Recombinant carp Il-4/13b1 (previously named Il-4/13B) was produced essentially as described previously^90^ and the expression plasmid^90^ a kind gift of Professor T. Moritomo and Dr. F. Katakura, Laboratory of Comparative Immunology, Nihon University. In short, the poly-His-tagged Il-4/13b1 protein was expressed in Rosetta-gami B (DE3) pLysS Competent cells (Novagen) and purified using sepharose beads (Qiagen) followed by gel chromatography size exclusion using Superdex 200 Prep Grade 26/600 column (GE Healthcare). Protein analysis by SDS-PAGE (12% Tris–HCl, Bio-Rad) stained with GelCode Blue Stain Reagent (Thermo Scientific) revealed that proteins were at least 95% pure and residual endotoxin content was shown to be < 0.005 EU/ml (EndoZyme II Recombinant Factor C (rFC) Assay, Hyglos GmbH).

### RNA extraction

Extracted RNA was used for Illumina sequencing and RT-qPCR experiments. For this, 1.5 × 10^6^ macrophages were polarized in 24-well plates (Corning) in a total volume of 450 µl/well and stimulated for 6 h before RNA extraction. Technical replicates were pooled, and total RNA was extracted from 3 × 10^6^ cells using the Rneasy mini kit (Qiagen) according to the manufacturer’s protocol including the on-column DNase digestion using the RNase-free DNase digestion kit (Qiagen). RNA was stored at -80ºC until use for sequencing and qPCR experiments.

### Illumina sequencing and sequencing analysis

Quality, integrity and quantity of the RNA was assessed using a Bioanalyzer (Agilent 2100 total RNA Nano series II chip, Agilent). RNAseq libraries were prepared from 0.5 μg total RNA using the TruSeq Stranded mRNA Library Prep kit according to the manufacturer's instructions (Illumina Inc.). All RNAseq libraries were sequenced on an Illumina HiSeq2500 sequencer as 1 × 50 nucleotides single-end reads according to Kolder et al.^108^ and Petit et al.^109^. The Illumina pipeline was used for image analysis and base calling. Reads were aligned to the genome assembly of common carp (BioProject: PRJNA73579)^108^. Secondary alignments of reads were excluded by filtering the files using SAMtools (version 0.1.18)^110^. Aligned fragments per predicted gene were counted from SAM alignment files using the Python package HTSeq (version 0.5.3p9)^111^.

### Differential gene expression

Differential gene expression was analyzed using the bioinformatics package DESeq 2.0 (v1.22.2) and R statistical software (3.5.5)^112^. Statistical analysis was performed using a paired design with unstimulated cells as control and performed for LPS, cAMP, LPS + Ifn-γ and Il-4/13b1 stimulated macrophages independently (*n* = 3 independent cultures for each stimulus). The paired design allowed for a better comparison between independent cultures, reducing noise generated by independent culture to culture differences. Within DESeq 2.0, *p*-values were adjusted using Benjamini & Hochberg corrections for controlling false discovery rate and results were considered statistically significant when *p*_adjusted_ ≤ 0.05. Additional subsetting and analysis was performed based on the log2 fold change (DESeq 2.0) and the number of reads per kilobasepair per million reads (RPKM). Proportional Venn diagrams were generated using the VennDiagram package^113^ (1.6.20) in R statistical software (3.5.5).

### Gene Ontology analysis

Gene Ontology (GO) analysis GO analysis of differentially expressed was performed with GOrilla^114,115^. Separate analyses were performed for differentially expressed genes [*F*_adjusted_ < 0.05 and upregulated (log2 foldchange > 1) or downregulated (log2 fold change < − 1)] for LPS (M1), cAMP (M2) and LPS + Ifn-γ-stimulated macrophages compared to unstimulated controls. Stable Ensembl zebrafish IDs were used for analysis and the full list of annotated common carp genes^108^ functioned as background list for the enrichment analysis. GO analysis required removal of duplicate Ensembl IDs and those IDs not associated with GO-terms in each dataset. GO terms were considered significantly enriched if False Discovery Rate (FDR) q-values ≤ 0.05. FDR q-values are *p* values corrected for multiple testing using the Benjamini and Hochberg (1995) method.

### Real-time quantitative PCR

RT-qPCR analysis was performed with a Rotor-Gene 6000 (Corbett Research) using ABsolute qPCR SYBR Green Mix (Thermo Scientific). The primers used are shown in Supplementary table 6. Fluorescence data from RT-qPCR experiments were analyzed using Rotor-Gene Analysis software (v1.7). The take-off value for each sample and the average reaction efficiencies (*E*) for each primer set were obtained upon Comparative Quantitation Analysis from Rotor Gene Software^116^. The relative expression ratio (*R*) of a target gene was calculated based on the average *E* and the take-off deviation of sample versus control and expressed relative to the *s11* protein of the *40 s* subunit as a reference gene.

### Statistical analysis

Raw data of technical replicates were averaged per individual before statistical analysis was performed using IBM SPSS Statistics 26. For RT-qPCR data, statistical analysis was performed on log-transformed data to obtain normal distributions. Significant differences between groups were determined using a (repeated measures) one-way analysis of variance (ANOVA) followed by Tukey’s post-hoc test for multiple comparisons. In absence of sphericity (Mauchly’s test of sphericity) in repeated measures ANOVA the Geisser-Greenhouse correction was applied. In case of unequal variances determined by Levene’s test, Dunnett’s T3 post-hoc test was used for multiple comparisons. In the absence of normality as determined by the Shapiro–Wilk test, the non-parametric Friedman’s two-way ANOVA by ranks was used for paired analysis and the Kruskal–Wallis test was used for independent samples.

## Supplementary information

Supplementary data.

Supplementary Table 1.

Supplementary Table 2.

## Data Availability

The datasets analyzed during the current study are available in the NCBI Sequence Read Archive, BioProject ID: PRJNA627088 (RNA sequencing data). Or available from the corresponding author upon reasonable request (other data).
