# Supplementary material for: Transcriptome sequencing supports a conservation of macrophage polarization in fish
Source: Sci Rep. 2020 Aug 10;10:13470. doi: 10.1038/s41598-020-70248-y (PMC7418020; doi:10.1038/s41598-020-70248-y)
Supplement: Supplementary file 1 — Supplementary data. [file 41598_2020_70248_MOESM1_ESM.pdf]

Supplementary data: Transcriptome sequencing supports a conservation of macrophage polarization in fish

Running title: conservation of macrophage polarization in fish

Annelieke S. Wentzel[1], Jules Petit[2], Wouter G. van Veen[3], Inge Rosenbek Fink[1], Marleen H. Scheer[1], M. Carla Piazzon[4], Maria Forlenza[1], Herman P. Spaink[5], Geert F. Wiegertjes[2*]

[1]Cell Biology and Immunology Group, [2]Aquaculture and Fisheries Group, [3]Experimental Zoology Group, Wageningen University & Research, De Elst 1, 6708 WD, Wageningen, Netherlands. [4]Fish Pathology Group, Institute of Aquaculture Torre de la Sal (IATS-CSIC), 12595 Ribera de Cabanes, Castellón, Spain. [5]Institute of Biology, Leiden University, Einsteinweg 55, 2332 CC, Leiden, Netherlands. [*]email: geert.wiegertjes@wur.nl

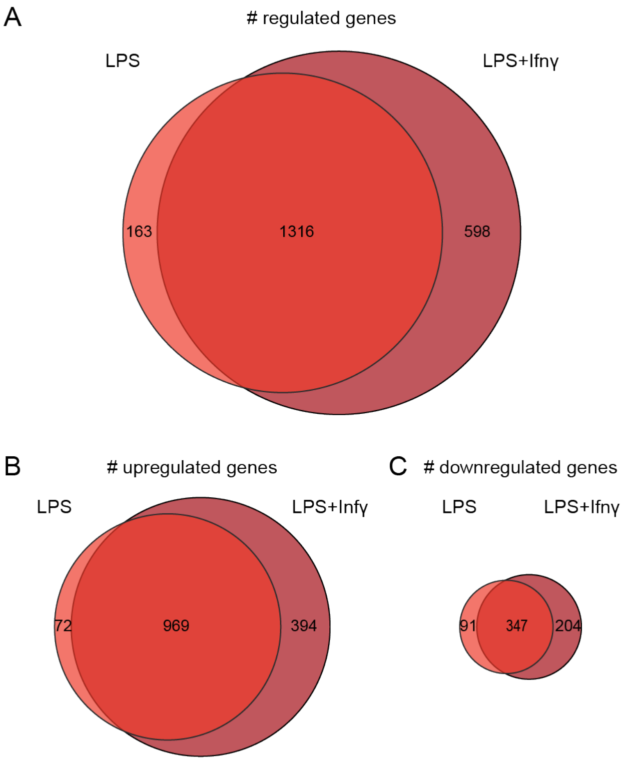

Supplementary figure 1: Addition of Ifn-γ to LPS stimulation induces largely similar transcriptional profiles in carp macrophages. Proportional Venn diagrams depicting transcriptional changes of carp macrophages polarized for 6 h with 30 µg/ml LPS with (LPS + Ifn-γ) or without (LPS) the addition of 100 ng/ml IFN-γ compared to unpolarized control macrophages. The total number of significantly ($p_{adjusted} < 0.05$) regulated genes (A) is further specified to show the number of genes upregulated (B) and downregulated (C). Data are of $n = 3$ fish.

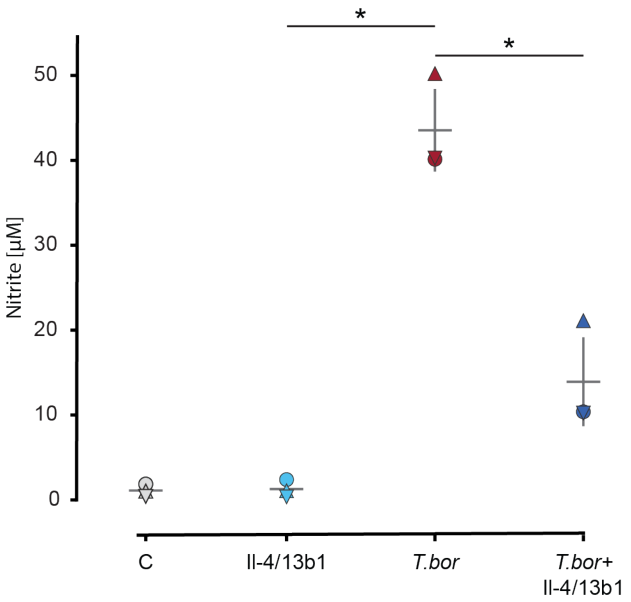

Supplementary figure 2: Bioactivity of recombinant Il-4/13b1 displayed as a downregulation of *T borreli* induced nitric oxide (NO) production. NO production of total mid-kidney leukocytes measured as nitrite concentration in culture supernatants after 96 h. Cells were kept as unstimulated controls (grey), or stimulated with Il-4/13b1 (light blue), *T. borreli* lysate (*T.bor*, red) or *T. borreli* and Il-4/13b1 (dark blue). Data of $n = 3$ fish were analyzed using a repeated measures ANOVA.

Supplementary tables 1 and 2 and associated captions are included in separate Excel spreadsheets.

Supplementary table 3: Transcriptional phenotype of carp LPS + Ifn-γ polarized macrophages is similar to LPS stimulated macrophages, with higher fold changes between stimulated and control cells. Genes most highly upregulated in M1 macrophages polarized with 30 µg/ml LPS + 100 ng/ml IFN-γ for 6 h in descending order. Genes were included when the following criteria were met: $p_{\text{adjusted}} < 0.05$ and average reads per kilobasepair per million reads (RPKM > 50) in stimulated or control samples. The 20 most highly upregulated genes (excluding paralogs) were depicted with the gene abbreviation (Gene), gene description, gene identifier (Gene ID cypCar), log2 fold change compared to unstimulated control macrophages (Log2FC), a short description of their main function (in macrophages if possible) and average RPKM in control (RPKM C) and LPS (RPKM LPS + Ifn-γ) polarized macrophages. Multiple cypCar IDs per gene were included if paralogs fell within the top 20 most upregulated genes. Each cypCar gene ID represents an individual gene unless cypCars are combined with a dash (–), indicating a (most likely) single gene that was wrongly predicted as two genes. Data are of $n = 3$ fish.

Gene	Gene description	Gene ID cypCar	Log2 FC	Main Function	RPKM C	RPKM LPS+ Ifn-γ
il12p35	Interleukin 12 subunit alpha (p35)	00024698–<br>00024699	11.0<br>9.6	P35 subunit of the pro-inflammatory cytokine Il-12. Involved in the activation of Th1 and NK cells[1].	0.1<br>0.4	261.4<br>263.7
il1β	Interleukin 1 beta	00043439–<br>00043440	8.4<br>8.2	Pro-inflammatory cytokine. Mediator of various cellular activities including proliferation, differentiation and apoptosis[2].	72.0<br>74.0	21098.7<br>18253.6
agrn	Agrin	00029572	7.3	Extracellular-matrix protein involved in monocyte/macrophage survival, cytoskeleton formation and phagocytosis[3].	32.7	2946.9
steap4	Six-transmembrane epithelial antigen of prostate 4	00042005	7.1	Metalloreductase involved in the transfer of ions from Fe_{3}^{+} and Cu_{2}^{+} to NAD and plays a role in cellular homeostasis during inflammation. Increased steap4 may reduce circulating iron available for parasites[4].	4.6	384.7
saa	Serum amyloid A protein	000373330<br>0036204	6.9<br>5.6	Acute phase protein, chemotactic to phagocytes and induces transcription of several pro-inflammatory cytokines[5,6].	2.8<br>77.6	270.3<br>2427.9
lacc1	Laccase-domain containing protein 1	00009189	6.2	Promotes fatty-acid oxidation, inflammasome activation, mitochondrial and NADPH-oxidase-dependent reactive oxygen species production and bactericidal activity of macrophages[7].	4.3	232.9
nos2b	Nitric oxide synthase 2b	00004424<br>00024539	5.9<br>5.9	Production of antimicrobial nitric oxide. Has functioned as M1 marker since macrophage polarization was described [8,9].	10.1<br>43.6	533.3<br>2183.6
ptgs2a/ cox2	Prostaglandin-endoperoxide synthase 2a	00026925	5.8	Also known as Cox-2. Increased expression in human M1 macrophages[10,11].	8.7	351.3
mhc2dbb	Major histocompatibility complex class II DBB	00014759	5.5	Classified as non-classical MHC II with conserved domains for CD4 binding[12,13]. No functional studies have been performed.	1.7	64.7

Gene	Gene description	Gene ID cypCar	Log2 FC	Main Function	RPKM C	RPKM LPS+ Ifn-γ
zgc:174917	*Uncharacterized protein*	*00021523*	5.4		1.9	68.1
mpeg1.2/ perforin 2	Macrophage-expressed 1/ Perforin 2	00002880	5.2	Pore forming perforin. Induced upon infection in zebrafish macrophages[14].	2.0	64.0
olfm4	Olfactomedin-4-like	00047183	5.1	Extracellular glycoprotein indicated in myeloid-specific differentiation and neutrophil inflammation[15–17]	1.8	51.7
tmem238	Transmembrane protein 238	00015661	5.0	Classified as transmembrane protein, no specific functions have been described.	2.4	59.1
mecr	Mitochondrial Enoyl-[acyl-carrier-protein] reductase	00002503<br>00002502	5.0<br>4.9	Protein involved in mitochondrial fatty acid synthesis. Increased upon *Salmonella enteritidis* infection in chicken macrophages[18].	13.6<br>24.9	302.0<br>568.4
il6	Interleukin 6	00035927	5.0	Pro-inflammatory cytokine produced by macrophages in response to PRR activation[19,20].	30.2	843.5
acod1/ irg1	Aconitate decarboxylase 1/ Immune responsive gene 1	00007903<br>00026281	4.9<br>4.9	Catalyzes the production of itaconate. High expression in mammalian M1 macrophages contributes to metabolic reprogramming[21,22].	71.5<br>6.4	1752.0<br>155.5
tdh	L-threonine dehydrogenase	00008269	4.7	Converts L-theonine into glycine. Glycine modulates macrophage activity[23,24].	16.3	343.5
il4i1	IL4 induced 1	00008491<br>00008492	4.5<br>4.4	Expressed by macrophages stimulated by microbial derived products. Exerts antibacterial activities.through the production of H_2O_2 and other toxic metabolites[25].	10.6<br>9.3	210.0<br>162.4
si:ch1073-67j19.1	Uncharacterized protein	00039673	4.5		314.3	6004.4
cygb1	Cytoglobin 1	00046202	4.3	Oxygen-carrying globin, expressed in macrophages and increased during oxidative stress. Protection mechanism against oxidative stress[26,27].	3.0	55.3

1. Tait Wojno, E. D., Hunter, C. A. & Stumhofer, J. S. The Immunobiology of the Interleukin-12 Family: Room for Discovery. *Immunity* **50**, 851–870 (2019).

2. Mantovani, A., Dinarello, C. A., Molgora, M. & Garlanda, C. Interleukin-1 and Related Cytokines in the Regulation of Inflammation and Immunity. *Immunity* **50**, 778–795 (2019).

3. Mazzon, C. *et al.* Agrin is required for survival and function of monocytic cells. *Blood* **119**, 5502–5511 (2012).

4. Scarl, R. T., Lawrence, C. M., Gordon, H. M. & Nunemaker, C. S. STEAP4: its emerging role in metabolism and homeostasis of cellular iron and copper. *J. Endocrinol.* **234**, R123–R134 (2017).

5. Badolato, R. *et al.* Serum amyloid A is a chemoattractant: induction of migration, adhesion, and tissue infiltration of monocytes and polymorphonuclear leukocytes. *J. Exp. Med.* **180**, 203 LP – 209 (1994).

6. He, R. L. *et al.* Serum amyloid A induces G-CSF expression and neutrophilia via Toll-like receptor 2. *Blood* **113**, 429 LP – 437 (2009).

7. Lahiri, A., Hedl, M., Yan, J. & Abraham, C. Human LACC1 increases innate receptor-induced responses and a LACC1 disease-risk variant modulates these outcomes. *Nat. Commun.* **8**, 15614 (2017).

8. Mills, C. D., Kincaid, K., Alt, J. M., Heilman, M. J. & Hill, A. M. M-1/M-2 Macrophages and the Th1/Th2 Paradigm. *J. Immunol.* (2000). doi:10.4049/jimmunol.164.12.6166

9. Nathan, C. F. & Hibbs, J. B. Role of nitric oxide synthesis in macrophage antimicrobial activity. *Curr. Opin. Immunol.* **3**, 65–70 (1991).

10. Martinez, F. O., Gordon, S., Locati, M. & Mantovani, A. Transcriptional Profiling of the Human Monocyte-to-Macrophage Differentiation and Polarization: New Molecules and Patterns of Gene Expression. *J. Immunol.* **177**, 7303–7311 (2006).

11. Jablonski, K. A. *et al.* Novel Markers to Delineate Murine M1 and M2 Macrophages. *PLoS One* **10**, e0145342 (2015).

12. Sambrook, J. G., Figueroa, F. & Beck, S. A genome-wide survey of Major Histocompatibility Complex (MHC) genes and their paralogues in zebrafish. *BMC Genomics* **6**, 152 (2005).

13. Dijkstra, J. M., Grimholt, U., Leong, J., Koop, B. F. & Hashimoto, K. Comprehensive analysis of MHC class II genes in teleost fish genomes reveals dispensability of the peptide-loading DM system in a large part of vertebrates. *BMC Evol. Biol.* **13**, 260 (2013).

14. Benard, E. L. *et al.* Macrophage-Expressed Perforins Mpeg1 and Mpeg1.2 Have an Anti-Bacterial Function in Zebrafish. *J. Innate Immun.* **7**, 136–152 (2015).

15. Zhang, J. *et al.* Identification and characterization of a novel member of olfactomedin-related protein family, hGC-1, expressed during myeloid lineage development. *Gene* **283**, 83–93 (2002).

16. Clemmensen, S. N. *et al.* Olfactomedin 4 defines a subset of human neutrophils. *J. Leukoc. Biol.* **91**, 495–500 (2012).

17. Alder, M. N. *et al.* Olfactomedin 4 marks a subset of neutrophils in mice. *Innate Immun.* **25**, 22–33 (2018).

18. Sekelova, Z. *et al.* Differential protein expression in chicken macrophages and heterophils in vivo following infection with Salmonella Enteritidis. *Vet. Res.* **48**, 35 (2017).

19. Mantovani, A. *et al.* The chemokine system in diverse forms of macrophage activation and polarization. *Trends Immunol.* **25**, 677–686 (2004).

20. Beyer, M. *et al.* High-Resolution Transcriptome of Human Macrophages. *PLoS One* **7**, (2012).

21. Van den Bossche, J., O'Neill, L. A. & Menon, D. Macrophage Immunometabolism: Where Are We (Going)? *Trends Immunol.* **38**, 395–406 (2017).

22. O'Neill, L. A. J. & Artyomov, M. N. Itaconate: the poster child of metabolic reprogramming in macrophage function. *Nat. Rev. Immunol.* **19**, 273–281 (2019).

23. Carmans, S. *et al.* The inhibitory neurotransmitter glycine modulates macrophage activity by activation of neutral amino acid transporters. *J. Neurosci. Res.* **88**, 2420–2430 (2010).

24. Loomis, W. P., den Hartigh, A. B., Cookson, B. T. & Fink, S. L. Diverse small molecules prevent macrophage lysis during pyroptosis. *Cell Death Dis.* **10**, 326 (2019).

25. Puiffe, M.-L., Lachaise, I., Molinier-Frenkel, V. & Castellano, F. Antibacterial Properties of the Mammalian L-Amino Acid Oxidase IL4I1. *PLoS One* **8**, e54589 (2013).

26. Li, D. *et al.* Cytoglobin Up-regulated by Hydrogen Peroxide Plays a Protective Role in Oxidative Stress. *Neurochem. Res.* **32**, 1375–1380 (2007).

27. Oleksiewicz, U., Liloglou, T., Field, J. K. & Xinarianos, G. Cytoglobin: biochemical, functional and clinical perspective of the newest member of the globin family. *Cell. Mol. Life Sci.* **68**, 3869–3883 (2011).

Supplementary table 4: Putative receptors and downstream signaling molecules of Il-4/13 are expressed in unstimulated macrophages. Putative receptor subunits and downstream signaling molecules were depicted with the gene abbreviation (Gene), gene identifier (Gene ID cypCar) and average RPKM in unstimulated control macrophages (RPKM C) of $n = 3$ fish.

**Gene**	**Gene ID cypCar**	**Average RPKM C**
il4r2	00012277	30
il13ra1	00023082	163
	00003131	29
il13ra2	00000342	30
	00011497	166
il2rga	00020372	583
	00009989	151
il2rgb	00003404	55
stat6	00003253	20
	00024537	28
stat3	00029051	95
	00030463	64
	00030464	33

Supplementary table 5: Marker candidates for M1 and/or M2. Candidate markers meeting the following requirements for M1 and/or M2 macrophages (indicated in bold); significantly ($p_{adjusted}$ < 0.05) up-or downregulated compared to unstimulated controls > 1.5 log2 or < -1.5 log2, difference in regulation between M1 and M2 > 2.5 log2 and average RPKM > 50 in the stimulated samples or unstimulated controls. Candidate markers depicted with the gene identifier (Gene ID cypCar), the gene abbreviation (Gene), log2 fold change (Log2FC) in M1 and M2 macrophages compared to unstimulated controls and average RPKM in M1 and M2 macrophages and unstimulated control macrophages in the corresponding dataset (C M1, C M2) of *n* = 3 fish for both M1 and M2 datasets. NA indicates an absence of log2FC calculations due to expression below the detection limit in one or multiple samples. Letters in superscript next to the gene abbreviation indicate a possible mis-annotation of a single gene as two separate genes.

Gene ID cypCar	Gene	Log2FC M1	RPKM C	RPKM M1	Log2FC M2	RPKM C M2	RPKM M2
**Marker candidates for M1 and M2 macrophages**							
Significantly upregulated in M1 and significantly downregulated in M2							
00046689	*marcksl1a*	1.7	155.2	432.1	-1.6	204.1	54.8
Significantly downregulated in M1 and significantly upregulated in M2							
00047225	*mrc1b*	-1.7	217.8	66.1	2.0	125.8	432.0
00023855	*mrc1b*	-1.6	192.2	60.6	2.0	119.1	393.9
00046879	*mrc1b*	-1.5	1188.3	369.0	1.7	615.0	1662.5
00034006	*mrc1b*	-1.5	1053.0	336.0	1.7	543.6	1454.6
00046880	*mrc1b*	-1.5	1590.7	527.2	1.6	857.0	2248.6
**Marker candidates for M1 macrophages alone**							
Significantly upregulated in M1 and significantly downregulated in M2							
00004424	*nos2b*	5.3	10.1	355.4	-0.7	25.5	13.3
00012414	*hsp70*[a]	3.2	55.0	479.5	-0.9	41.3	16.9
00012415	*hsp70*[a]	3.2	43.5	393.8	-0.9	34.9	14.2
00000280	*hsp70*	3.1	21.7	188.8	-0.8	15.3	6.9
00030534	*hsp70*	3.1	50.5	397.1	-0.8	36.1	15.9
00048796	*hsp70*	3.0	27.1	221.0	-0.8	20.3	9.8
00041000	*cfb/c2-a3*	2.3	16.4	71.1	-0.6	23.6	12.4
00005739	*cfb/c2-a3*	2.3	240.3	1002.5	-0.5	329.3	189.6
00032673	*dnajb1b*	2.1	18.5	70.3	-1.4	14.5	4.7
00045205	*f3b*	2.0	72.9	229.3	-0.8	62.1	29.4
00033031	*malt3*	1.9	23.8	80.8	-1.1	39.5	17.0
Significantly upregulated exclusively in M1							

Gene ID cypCar	Gene	Log2FC M1	RPKM C	RPKM M1	Log2FC M2	RPKM C M2	RPKM M2
00024698	*il12p35*[b]	8.8	0.1	56.0	NA	0.18	1.53
00024699	*il12p35*[b]	7.2	0.4	59.4	NA	0.27	1.85
00037333	*saa*	6.3	2.8	183.8	0.7	4.1	5.8
00024539	*nos2b*	5.2	43.6	1427.6	-0.4	102.4	62.9
00046202	*cygb1*	4.3	3.0	56.4	0.3	21.9	20.2
00002926	*cxcl13c*	4.0	8.6	96.4	0.3	6.4	6.8
00016657	*cxcl8l1*	4.0	230.4	2984.1	0.3	98.1	99.4
00008491	*il4i1*	3.5	10.6	106.3	0.2	6.1	6.2
00033150	*il1r2*	3.5	15.2	137.9	0.0	14.4	12.5
00015036	*slc2a6*	3.4	29.0	267.1	0.1	61.6	60.7
00024970	*il1r2*	3.4	70.1	626.2	0.2	63.6	62.4
00018821	*ptges*	3.4	7.7	68.3	0.2	9.2	8.6
00008492	*il4i1*	3.4	9.3	85.3	0.1	5.1	4.6
00017325	*igsf6*	3.4	17.3	164.2	0.2	26.1	25.5
00007848	*blvrb*	3.3	8.7	76.1	0.2	13.0	12.7
00002648	*uncharac-terized prot.*	3.1	18.5	134.4	0.2	19.5	18.1
00028558	*mettl17*	3.1	11.4	82.8	0.2	7.8	7.6
00028560	*pf1*	2.7	67.6	346.2	-0.2	50.0	33.6
00032122	*flot1b*	2.5	45.8	229.7	-0.4	59.7	38.8
00028528	*tnfsf13b*	2.4	39.8	181.4	-0.5	33.4	20.4
00040937	*pak1*	2.1	13.9	50.1	-0.6	22.9	13.5
Significantly upregulated in both M1 and M2 but at least 2.5 log2 higher in M1 compared to M2							
00043439	*il1b*[c]	7.6	72.0	12832.1	4.2	46.1	945.4
00043440	*il1b*[c]	7.5	74.0	11482.7	4.0	42.2	771.8
00042005	*steap4*	7.0	4.6	405.9	3.8	6.8	100.6
00029572	*agrn*	7.0	32.7	2569.8	4.3	11.2	153.8
00026925	*ptgs2a*	5.7	8.7	319.8	2.8	5.7	33.6
00047183	*olfm4*	5.4	1.8	69.5	1.7	8.2	23.5
00036204	*saa*	5.3	77.6	1968.3	1.4	83.1	192.6
00002503	*mercr*[c]	5.1	13.6	340.7	0.9	16.4	24.8
00002502	*mercr*[c]	5.0	24.9	627.8	1.0	29.4	46.7
00035927	*il6*	5.0	30.2	864.5	1.7	32.3	80.6
00007903	*irg1*	4.6	71.5	1404.8	1.9	129.3	406.7
00026281	*irg1*	4.5	6.4	121.8	1.9	12.2	37.3
Downregulated exclusively in M1							
00017566	*dab2*	-2.8	91.2	12.7	0.1	71.8	63.4

Gene ID cypCar	Gene	Log2FC M1	RPKM C	RPKM M1	Log2FC M2	RPKM C M2	RPKM M2
Significantly downregulated in M1 and significantly upregulated in M2							
00002074	*dab2*	-1.9	123.1	31.3	0.6	96.0	125.6
00008536	*fgl2*	-1.9	468.9	107.3	0.8	365.6	529.5
00028026	*ca9 or ca14*	-1.8	55.3	14.1	0.9	42.7	65.7
**Marker genes for M2 macrophages alone**							
Significantly upregulated in M2 and significantly downregulated in M1							
00031163	*tcima*	-0.9	138.7	62.0	3.1	86.7	556.2
00027636	*rnf182*	-0.6	147.3	78.8	2.9	25.2	143.1
00001546	*glud1a*	-0.3	57.8	39.9	2.7	66.1	364.9
00016733	*tinagl1*	-1.5	7.0	2.3	2.6	22.0	113.1
00019572	*uncharac-terized prot.*	-0.5	28.4	16.7	2.5	32.4	142.2
00012277	*il4r.2*	-0.5	73.6	42.8	2.5	63.3	298.5
00000704	*entpd1*	-1.1	45.3	18.4	2.2	36.6	140.7
00037861	*mafbb*	-1.1	188.8	81.1	1.9	135.4	434.8
00014545	*pld3*	-1.2	26.5	9.9	1.9	23.2	72.6
00023082	*il13ra1*	-0.7	408.4	220.5	1.9	228.9	699.4
00001348	*hip1*	-1.0	60.1	25.9	1.7	44.9	117.0
00020333	*zfp36l1a*	-1.3	33.9	12.0	1.6	32.9	82.9
Significantly upregulated exclusively in M2							
00030755	*timp2b*	0.5	30.7	36.1	8.0	31.1	6476.0
00034483	*tgm2b*[d]	0.5	5.9	7.2	7.4	6.0	784.6
00030329	*tgm2b*[d]	0.8	4.8	6.9	6.9	6.4	698.7
00041907	*tgm2b*	-0.2	1.1	0.9	5.0	1.7	68.2
00024882	*pde4bb*	0.4	22.5	23.7	4.3	17.4	284.8
00035942	*angptl4*	-0.3	53.9	37.8	4.1	20.1	260.6
00049924	*angptl4*	-0.4	52.1	33.0	3.9	20.6	244.3
00018981	*tgm1l1*	0.9	1.3	2.0	3.8	45.6	509.5
00009477	*crema*	0.0	12.0	10.3	3.7	10.0	108.6
00020192	*pde4bb*	-0.1	37.4	29.3	3.6	25.4	266.8
00018013	*gadd45aa*	0.3	46.9	49.9	3.5	23.9	220.8
00040271	*rbp2a*	0.0	12.2	11.3	3.3	35.4	287.9
00035774	*vegfab*	-0.2	25.1	18.1	3.0	12.0	82.0
00015586	*rab26 or rab37*	-0.4	8.9	6.0	3.0	9.1	58.9
00043137	*cremb*	0.0	20.7	17.4	2.9	12.6	82.0

Gene ID cypCar	Gene	Log2FC M1	RPKM C	RPKM M1	Log2FC M2	RPKM C M2	RPKM M2
00040375	*crema*	0.1	40.2	36.6	2.9	31.1	197.2
00005288	*map3k15*	-0.2	25.6	19.6	2.8	12.8	77.0
00032256	*hsd11b2*	0.0	17.9	13.9	2.7	35.2	167.4
00011045	*soat1*	0.1	18.7	17.5	2.7	17.6	95.5
00005655	*btg1*	-0.2	119.3	87.8	2.7	77.8	426.6
00011046	*soat1*	0.0	19.6	16.9	2.7	18.3	99.5
00018053	*pnp5a*	0.3	617.8	608.5	2.6	404.4	2060.3
00007693	*eif4ebp3*	-0.1	241.5	189.8	2.6	192.3	950.2
00000437	*eif4ebp3*	-0.3	23.6	15.8	2.2	22.0	93.2
00032368	*tcim*	-0.5	60.3	37.3	2.2	24.8	100.0
Significantly upregulated in both M2 and M1 but at least 2.5 log2 higher in M2 compared to M1							
00001309	*cyr61l1*	3.1	0.5	4.6	9.4	0.2	103.8
00022158	*ramp2*	2.0	2.3	7.8	6.4	1.8	100.9
00035581	*dfna5a*	2.0	7.2	29.7	5.1	12.8	421.8
00034978	*arg2*	1.0	54.6	95.7	4.8	19.9	445.2
00015701	*csrnp1a*	0.9	18.8	31.0	4.2	7.5	117.1
00033214	*cremb*	1.0	24.7	41.7	3.6	15.6	164.5
00025413	*adam28*	0.8	112.9	166.6	3.4	114.1	1016.7
00024444	*lox*	0.4	34.2	38.8	3.2	12.0	83.9
Downregulated exclusively in M2							
00023172	*cxcl11-1*	0.0	90.7	85.4	-2.9	92.7	9.2
00002590	*cxcl11-like or cxcl18b*	-0.1	1429.4	1135.0	-2.7	1257.8	163.7
00023915	*f3b*	0.2	195.0	197.5	-2.3	151.0	23.7
Significantly downregulated in M2 and significantly upregulated in M1							
00038694	*tm4sf18*	0.5	35.1	42.0	-3.2	90.4	9.3
00027661	*pfkfb3*	0.9	40.9	63.7	-2.0	29.1	6.1
00021651	*cxcr3.3*	1.1	31.1	54.8	-2.0	53.0	10.1
00035137	*marcksl1a*	1.1	190.1	352.9	-1.6	244.1	67.2
Significantly downregulated in both M2 and 2 but at least 2.5 log2 lower in M2 compared to M1							
00048576	*tm4sf1*	-3.2	61.4	5.8	-0.5	59.9	37.2

Supplementary table 6: Primer sequences recognizing multiple paralogs of one gene that were used for RT-qPCR. Primer sequences are depicted with the gene abbreviation (Gene), Forward (FW) primer sequence, reverse (RV) primer sequence and Genbank accession number. [a]cypCar numbers identify open reading frames in the draft carp genome (BioProject: PRJNA73579) which were confirmed by RNA sequencing

Gene	**FW primer (5'-3')**	**RV primer (5' to 3')**	**Genbank acc. number[a]**
40s	*CCGTGGGTGACATCGTTAC*	*TCAGGACATTGAACCTCACTGTC*	AB012087
il1b	*AAGGAGGCCAGTGGCTCTGT*	*CCTGAAGAAGAGGAGGCTGTCA*	AJ245635
nos2	*AACAGGTCTGAAAGGGAATCCA*	*CATTATCTCTCATGTCCAGAGTCTCTTCT*	AJ242906
saa	*CCCAGGACAAGCCATTG*	*GCAGCATCATAGTTCCC*	cypCar_00037333 cypCar_00036204
timp2b	*TGGCAAAAAGGAATACCTG*	*CTCAGAGACTCCCAAGATTC*	cypCar_00030755 cypCar_00034223
tgm2b	*GCCTGGTATTTTGGACAGT*	*GCACTCAGCACTCTTGT*	cypCar_00030329- cypCar_00034483 cypCar_00041907
arg2	*GGAGACCTGGCCTTCAAGCATCT*	*TGATTGGCACGTCCAACT*	AJ618955
